# Medial Pulvinar Stimulation in Temporal Lobe Epilepsy: A Literature Review and a Hypothesis Based on Neuroanatomical Findings

**DOI:** 10.7759/cureus.35772

**Published:** 2023-03-05

**Authors:** Theodosis Kalamatianos, Georgios Mavrovounis, Panagiotis Skouras, Dionysios Pandis, Konstantinos Fountas, George Stranjalis

**Affiliations:** 1 Department of Neurosurgery, National and Kapodistrian University of Athens, Athens, GRC; 2 Department of Neurosurgery, Faculty of Medicine, University of Thessaly, Larissa, GRC; 3 1st Department of Neurology, Eginition Hospital, National and Kapodistrian University of Athens, Athens, GRC

**Keywords:** temporo-pulvinar bundle of arnold, inferior thalamic peduncle, temporal lobe epilepsy, medial pulvinar stimulation, deep brain stimulation

## Abstract

While bilateral stimulation of the anterior thalamic nuclei remains the only approved deep brain stimulation (DBS) option for focal epilepsy, two additional thalamic targets have been proposed. Earlier work indicated the potential of centromedian thalamic nucleus stimulation with recent findings highlighting the medial pulvinar nucleus. The latter has been shown to exhibit electrophysiological and imaging alterations in patients with partial status epilepticus and temporal lobe epilepsy. On this basis, recent studies have begun assessing the feasibility and efficacy of pulvinar stimulation, with encouraging results on the reduction of seizure frequency and severity. Building on existing neuroanatomical knowledge, indicating that the medial pulvinar is connected to the temporal lobe via the temporopulvinar bundle of Arnold, we hypothesize that this is one of the routes through which medial pulvinar stimulation affects temporal lobe structures. We suggest that further anatomic, imaging, and electrophysiologic studies are warranted to deepen our understanding of the subject and guide future clinical applications.

## Introduction and background

Epilepsy is one of the most common chronic neurological disorders affecting around 50 million people globally, according to the World Health Organization (WHO) [1]. It causes spontaneous, recurrent seizures and it is routinely managed with various antiseizure medications [2]. Despite the introduction of new antiepileptic medication, and even with proper, combination pharmacological therapy, nearly one-third of the patients does not achieve adequate seizure control, suffering from what is termed refractory epilepsy [3]. Refractory epilepsy has been shown to increase morbidity and mortality, and to significantly affect the cognition and the quality of life of patients [4]. It is, thus, imperative to identify new approaches in the treatment of refractory or drug-resistant epilepsy.

Deep brain stimulation (DBS) is a minimally invasive neurosurgical treatment modality involving the implantation of a pulse generator and its electrodes in order to modulate the electrical activity of pathological brain circuits in a controlled manner [5]. Initially used for the treatment of Parkinson’s disease, it has since become an established therapeutic option for an increasing number of disorders, including drug-resistant epilepsy [5]. Various cortical and subcortical structures have been suggested as potential direct and/or indirect targets of DBS for epilepsy, including the thalamus, the hippocampus, the cerebellar nuclei, and cortical zones [5,6]. The potential of white matter/axonal stimulation as an alternative to grey matter/nuclear stimulation in the treatment of epilepsy has also been discussed [7].

## Review

Methods

**Figure 1:**
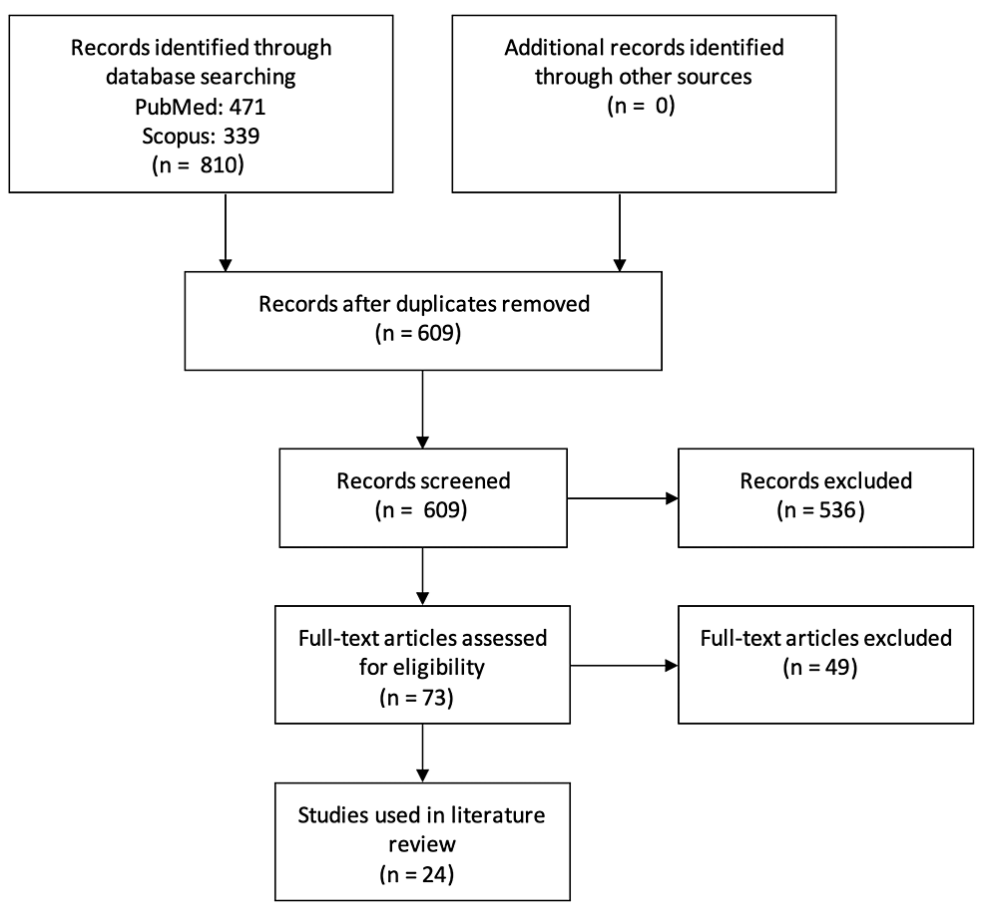
Flowchart illustrating the database search process.

This narrative review was based on electronic database searches of the PubMed and Scopus databases to identify relevant original articles and reviews. Only articles written in English were considered. The following algorithms were used in PubMed: (1) ("temporopulvinar" OR "inferior thalamic peduncle") and (2) ({"pulvinar" OR "medial pulvinar"} AND {"epilepsy" OR "stimulation"}). Similarly, the algorithms used in Scopus were as follows: (1) TITLE-ABS ("temporopulvinar" OR "inferior thalamic peduncle") and (2) TITLE-ABS ("pulvinar" OR "medial pulvinar") AND TITLE-ABS ("epilepsy" OR "stimulation"). Figure 1 illustrates the process that was followed to identify relevant articles. The last database search was performed on January 15, 2023.

Thalamic stimulation for epilepsy: earlier and recently investigated nuclear targets

Bilateral stimulation of the anterior thalamic nuclei for focal epilepsy remains the only approved DBS thalamic target. Based on the success reported from initial small, uncontrolled studies [8,9], a multicentre, double-blind (blinded period of three months), randomized-controlled trial (RCT) with 108 patients was conducted, showing that bilateral anterior thalamic nucleus (ATN) DBS resulted in reduction of seizure frequency and severity in patients with medically intractable partial and secondarily generalized seizures [10]. The stimulation of the anterior nucleus of the thalamus for epilepsy (ANTE) study [10] and its five-year efficacy and safety follow-up report [11] led the U.S. Food and Drug Administration (FDA) to approve bilateral ATN DBS for partial (focal onset) drug-resistant epilepsy [5,10-12].

Aside from the ATN, the potential of two additional thalamic nuclei as DBS targets for epilepsy has been investigated in previous studies, the centromedian thalamic nucleus (CMT) with more recent findings drawing attention to the medial pulvinar nucleus. In a similar fashion to ATN DBS, researchers in the 1980s studied the potential of CMT DBS for epilepsy. Following the publication of promising reports [13], two RCTs were conducted in 1992 and 2000 showing some success for patients with Lennox-Gastaut syndrome and generalized tonic-clonic seizures [14,15]. Since then, CMT DBS has been investigated in multiple studies [16-18]. Notably, the electrical stimulation of thalamic centromedian nucleus for epilepsy of Lennox-Gastaut syndrome (ESTEL) trial, reported a reduction of electrographic seizures in patients with Lennox-Gastaut after CMT DBS [16]. Recently, a retrospective case series reported promising results with the combination of CMT and ATN DBS for the management of patients with generalized, multifocal, posterior origin and poorly localized drug-resistant epilepsy [17].

In the remainder of the present review, we provide an overview of the increasing evidence implicating the medial pulvinar nucleus in temporal lobe epilepsy (TLE) and its treatment via DBS of this nucleus [19]. Moreover, we propose a hypothesis regarding the anatomical circuitry mediating these effects and highlight additional research that is warranted on this topic.

The pulvinar nucleus: anatomofunctional considerations

Pulvinar Parcellation

The pulvinar nucleus is the largest thalamic nucleus and is part of the lateral thalamic nuclei [20]. According to the cytoarchitecture-based human anatomical thalamic atlas of Morel et al., the pulvinar nucleus consists of four distinct nuclei, namely the medial, the inferior, the lateral, and the anterior/oral pulvinar nuclei [20]. In recent years, parcellations of individual brain regions achieve greater accuracy when combinations of techniques are implemented, with integration of cytoarchitecture, function, and connectivity [21]. A functional parcellation scheme, based on a meta-analysis of task-related functional MRI studies including around 30,000 subjects, has divided the human pulvinar into five clusters (anterior, superior, inferior, medial, and lateral), based on distinct coactivation with other brain areas [21]. The medial pulvinar, as defined by Morel et al., did not correspond to a specific cluster, rather it was represented in all clusters, with higher percentages in the medial, inferior, and superior clusters [20]. As the authors noted, this was to be expected because the cytoarchitecturally-defined Morel medial pulvinar is the largest pulvinar subnucleus and is likely to be functionally non-homogenous. The smaller pulvinar subnuclei demonstrated better correlation between the Morel atlas and the functional parcellation scheme. The anterior and inferior Morel subnuclei corresponded almost perfectly to the anterior and inferior clusters as defined by the meta-analytic method, while the lateral Morel subnucleus was divided into the inferior and lateral clusters [21]. Similar findings regarding functional pulvinar parcellation were reported by a recent meta-analysis of resting-state functional MRI studies by Guedj et al. [22]. The authors identified five distinct pulvinar subdivisions (anterior, inferior, lateral, dorsomedial, and ventromedial). The Morel anterior and inferior pulvinar nuclei matched the anterior and inferior subnuclei almost completely, while the inferior and lateral subdivisions corresponded to the Morel lateral pulvinar nucleus. Finally, the Morel medial pulvinar nucleus included parts of all subdivisions.

Pulvinar Connectivity and Function in Humans and Non-human Primates

The pulvinar nucleus demonstrates extensive connectivity with various cortical and subcortical regions in both non-human primates and humans. In keeping with primate studies, a tractography study of normal volunteers by Leh et al. illustrated connections between the pulvinar and various other brain structures, such as the caudate, the superior colliculus, and the prefrontal, parietal, temporal, and occipital regions [23]. Functionally, studies on non-human primates, showed that the medial pulvinar has extensive connectivity with the prefrontal/frontal and temporal cortices, regions with important roles in cognition (mainly attention) and visuomotor/auditory integration [24-26]. The inferior and the lateral pulvinar subnuclei on the other hand, exhibit extensive connectivity with the occipital lobe, thus representing important visual processing regions [24]. When it comes to the human pulvinar, the anterior and lateral clusters (as identified by the meta-analysis by Guedj et al. mentioned in the "Pulvinar Parcellation" section above) have been shown to be involved in the domains of attention and action, while the dorsomedial and ventromedial clusters have been identified as parts of emotional and salience networks. The inferior cluster has been shown to be involved in memory and perception processes [22]. Finally, imaging studies have indicated pulvinar alterations in patients with schizophrenia [27], spatial neglect [28], and attention deficit hyperactivity disorder, among others [29].

The human medial pulvinar

Human Cadaveric Studies and Medial Pulvinar

There is a paucity of human cadaveric data on the connections displayed by the pulvinar in general and the medial pulvinar nucleus in particular. Using the Klingler technique of white matter dissection, Klingler and Gloor in 1960 provided an anatomical description of two fiber pathways connecting the anterior temporal lobe and the thalamus, namely the extracapsular thalamic peduncle and the temporopulvinar bundle of Arnold [30]. The extracapsular thalamic peduncle connects the medial thalamus with the anterior temporal cortex, passing through the ansa peduncularis, and has an entirely extracapsular course [30,31]. According to Klingler and Gloor in 1960, the temporopulvinar bundle of Arnold has, in contrast, an intracapsular course and is part of what should be called the inferior thalamic peduncle (ITP). The temporopulvinar bundle of Arnold, and consequently the ITP, connects the pulvinar with the anterior temporal region, reaching the temporal pole [30,31].

As noted by Klingler and Gloor in 1960, and more recently by Serra et al., some authors have inappropriately used the term ITP for the description of the extracapsular thalamic peduncle [30,31]. Advocating the nomenclature suggested by Klingler and Gloor in 1960, Serra et al. proposed that the terms inferior, superior, anterior, and posterior thalamic peduncles should be reserved for thalamic radiations passing through the internal capsule towards the tip of the temporal lobe, the anterior parietal cortex/central region, the frontal cortex, and the posterior parietal/occipital cortex, respectively [31]. It is worth mentioning that, while Klingler and Gloor in 1960 termed the bundle connecting the pulvinar with the anterior temporal lobe as “temporopulvinar bundle of Arnold” and suggested that it is part of the ITP, Serra et al. only mentioned the term ITP to describe the bundle connecting the same regions. Importantly, Klingler and Gloor in 1960, note that based on previous anatomical and electrophysiological data, the connections between the anterior temporal cortex and the pulvinar contain both ascending and descending fibers [30].

More recently, Weiss et al. investigated the temporopulvinar bundle of Arnold in an additional fiber dissection study [32]. The bundle’s trajectory from its origin within the anterior temporal cortex was shown to be a posteromedial one, above/within the roof of the temporal horn, and below the tail of the caudate nucleus. Of note, the authors indicated that prior to continuing within the sublenticular segment of the internal capsule towards the pulvinar, the bundle provided connectivity with the lateral amygdala.

Tractography and Medial Pulvinar

A 2014 tractographic study, describing the temporopulvinar bundle of Arnold, presented some interesting findings [33]. Similarly to macaque monkeys, the bundle was shown to project to the medial and the anterior temporal cortex [34]. Another notable finding of the study by Nishio et al. was that the bundle originated from the anterior thalamus passed posteriorly through the pulvinar and then turned anteriorly at the temporal stem to reach the medial and anterior temporal lobes [33]. To our knowledge, this is the only study in humans that indicates an anterior thalamic origin of the temporopulvinar bundle of Arnold. Nevertheless, the authors of this study indicated the potential for an artifact of fiber tracking and suggested the need for further neuroanatomical investigations [33].

Stereo-Electroencephalography and Medial Pulvinar

In 2009, Rosenberg et al. utilized the electrodes implanted for diagnostic stereo-electroencephalographic (SEEG) exploration in patients with epilepsy to study the functional connectivity between the cerebral cortex and the medial pulvinar [35]. Indeed, they identified extensive reciprocal connectivity between the cortex and the medial pulvinar [35]. When the nucleus was stimulated, cortical evoked potentials were recorded in the temporal neocortex, the temporoparietal junction, the insula and the frontoparietal operculum with a frequency of 78% or more. Medial pulvinar evoked potentials were identified with a frequency of 76% or more when stimulation was applied to temporal neocortex structures to mesial temporal cortex structures and to the posterior cingulate cortex [35]. A similar study from the same institution confirmed the presence of a connection between the hippocampus and the medial pulvinar [36].

The Medial Pulvinar Nucleus in Temporal Lobe Epilepsy: Electrophysiologic and Imaging Studies

Several studies indicating various electrophysiologic and imaging changes of the medial pulvinar nucleus in patients with TLE and (partial) status epilepticus (SE), implicate this nucleus in epilepsy [37,38].

The involvement of the medial pulvinar nucleus in epilepsy has been indicated as early as 2006 by Guye et al. [39] and Rosenberg et al. [38]. Rosenberg et al. reported ictal alterations in the activity of the medial pulvinar nucleus by analyzing 74 seizures of mesial (amygdala, hippocampus, parahippocampal gyrus) and neocortical temporal origin in 14 patients undergoing SEEG presurgical assessment [38]. Medial pulvinar-associated ictal activity was observed in 80% of seizures [38]. Similarly, Guye et. al observed thalamic (mainly medial pulvinar) involvement during the course of temporal lobe seizures [39]. The medial pulvinar nucleus has also been shown to be involved in loss of consciousness and seizure termination in patients with temporal lobe epilepsy [40,41]. Finally, in line with the reciprocal connectivity reported by Rosenberg et al., Pizzo et al. identified high thalamic (42/74 of patients had electrodes exploring the pulvinar) epileptogenicity in patients with insulo-opercular epilepsy undergoing SEEG evaluation [35,42].

A plethora of MRI and single photon emission computed tomography (SPECT) studies have previously indicated pulvinar-related alterations, mainly in patients with TLE and partial SE [43-47]. MRI signal hyperintensities have been observed in the pulvinar of those patients, using diffusion-weighted (DWI), T2-weighted, and fluid-attenuated inversion recovery (FLAIR) MRIs [43,44,46]. Importantly, a study in SE patients reported that pulvinar MRI alterations were associated with temporal origin of the seizures [43]. Furthermore, a recent study using structural and diffusion MRI in healthy participants and patients with medial TLE illustrated dense connections between the medial pulvinar and the hippocampus [47]. Additionally, they reported decreased thalamic-connected volume in the pathways between the thalamus and the hippocampus of the medial TLE patients when compared with healthy volunteers [47]. A recent resting-state functional MRI study by Jo et al., comparing the functional connectivity of individual thalamic nuclei between patients with medial TLE and healthy volunteers, reported increased medial and anterior pulvinar connectivity in the epilepsy patient group [48]. Additionally, it has been reported that in TLE patients the pulvinar and anterior thalamus are highly susceptible to functional connectivity impairment [49]. Finally, the medial pulvinar has been found to be significantly less involved in SE originating in the frontal or parietal lobes [46].

Pulvinar stimulation in epilepsy

Based on previous evidence implicating the pulvinar in TLE, as well as the premise that the nucleus can be stereotactically reached with relative ease, recent studies have begun assessing the feasibility and efficacy of medial pulvinar stimulation for epilepsy [19,50].

In 2019, Filipescu et al. recruited eight patients with drug-resistant, focal TLE who underwent diagnostic SEEG exploration [19]. The recorded seizures were induced by stimulation of the hippocampus, and eligible patients were those who had at least one electrode in the medial pulvinar. The authors analyzed 19 seizures and reported a faster recovery in cases of high frequency (130 Hz, pulse width: 450 μs, duration: 3-7 seconds, current: 1-2 mA) medial pulvinar stimulation. Furthermore, they noted that none of the seizures with medial pulvinar stimulation presented secondary generalization. Finally, they reported a shorter tonic phase when medial pulvinar stimulation was applied. Overall, they concluded that the medial pulvinar nucleus is a safe and effective DBS target for TLE and that treatment resulted in less severe seizures, especially in relation to the altered level of consciousness [19]. In the same patient cohort, they identified that medial pulvinar stimulation leads to lower synchrony levels in those with improved levels of consciousness [51].

More recently, Burdette et al. used brain-responsive corticothalamic pulvinar stimulation in three patients with posterior quadrant, drug-resistant epilepsy; two of the patients had epileptogenic zones in the temporoparietal and temporooccipital regions and underwent electrode placement in the pulvinar [50]. Both patients responded to pulvinar stimulation (frequency: 125 Hz, charge density: 0.5 µC/cm^2^, duration: 2-5 seconds) with >50% seizure reduction, while one of the patients exhibited a 90% seizure reduction. The authors did not report any adverse events related to the stimulation.

Currently, a pilot, single-group, open-label study is recruiting patients with focal/multifocal drug-resistant epilepsy in order to evaluate the effect of medial pulvinar stimulation in the number of seizures (NCT04692701).

A hypothesis on the circuitry mediating medial pulvinar stimulation in epilepsy

**Figure 2:**
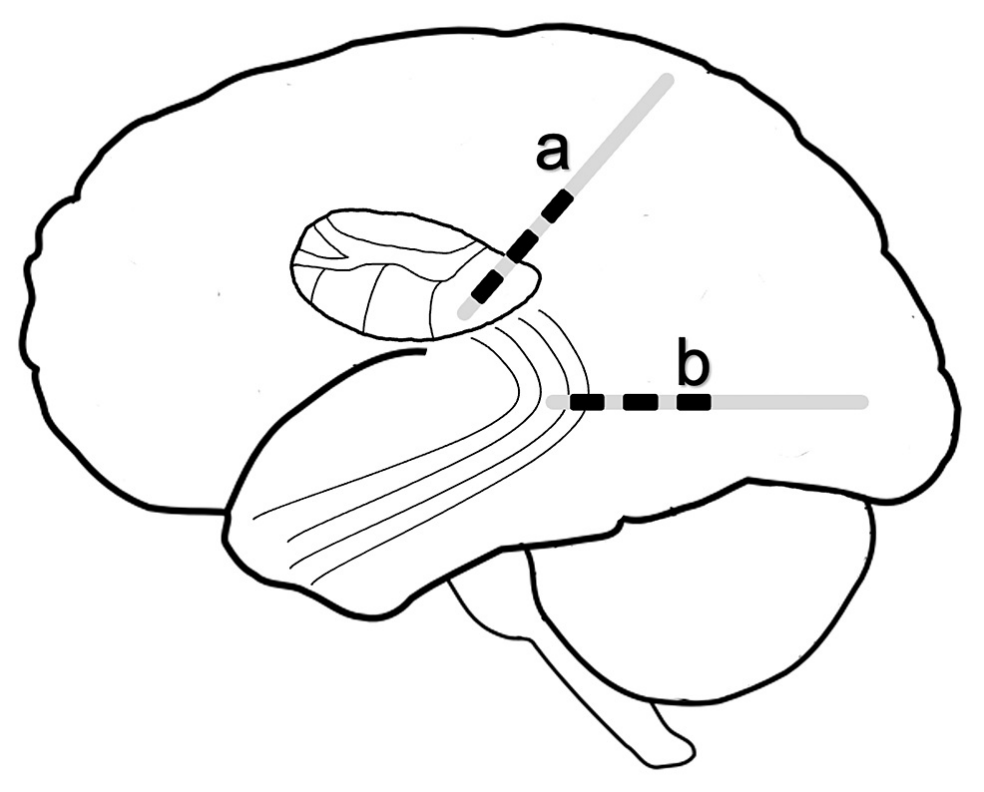
Pulvinar deep brain stimulation for temporal lobe epilepsy. The image is created by the authors of this study. The medial pulvinar (a) is a deep brain stimulation target currently under investigation. The temporopulvinar bundle of Arnold (b) represents a hypothetical alternative target.

Based on the present anatomical knowledge regarding the connectivity of the pulvinar complex and by analogy to the notion that the Papez circuit is of significance for the neuromodulation achieved via bilateral ATN DBS in drug-resistant, partial epilepsy, it can be postulated that the temporopulvinar bundle of Arnold represents an important direct route via which medial pulvinar stimulation can affect temporal lobe epilepsy and SE of temporal origin [5]. In this context, it is worth noting that recent studies have suggested that DBS for epilepsy directed at specific white matter tracts warrants further investigation [52,53]. As a corollary, the temporopulvinar bundle of Arnold, with its bidirectional connections between the medial pulvinar and the anterior and medial temporal lobe, represents a potential target of white matter-based DBS (Figure 2). This effort could be mediated by the introduction of directional leads which allow for more precise stimulation [54].

## Conclusions

In conclusion, we believe that medial pulvinar stimulation is a promising nuclear DBS target for refractory temporal lobe epilepsy and, similarly, the temporopulvinar bundle of Arnold might constitute an important white matter target. To further strengthen our understanding of the topic, we suggest that additional research is warranted on the following fronts. White matter cadaveric and imaging-based tractography studies could further elucidate the structural connectivity of the temporopulvinar bundle of Arnold. Moreover, the extent of overlap between structural and functional networks engaging the medial pulvinar nucleus could also be assessed during nuclear or white matter stimulation. In addition, electrophysiological studies could entail the study of (a) the effects of medial pulvinar stimulation on the electrical signals running through the temporopulvinar bundle of Arnold and (b) the medial pulvinar and the temporopulvinar bundle of Arnold in the context of neurostimulation for epilepsy of temporal origin.
